# The use of diagnostic tools to assess the risks of chemicals to freshwater ecosystems: towards a unified evaluation framework

**DOI:** 10.1007/s00267-025-02265-4

**Published:** 2025-09-17

**Authors:** Andreu Rico, Udo Hommen, Beate I. Escher, Alina Koch, Anne Bado-Nilles, Belén González-Gaya, Enya Cody, Francisco Sylvester, Gabriele Treu, Gastón Alurralde, Henner Hollert, Iker Alvarez-Mora, S. Jannicke Moe, Joanne de Jonge, Kelsey Ng, Manu Soto, Matthias Liess, Melis Muz, Mirco Bundschuh, Naroa Lopez-Herguedas, Nicolas Pucheux, Nikiforos Alygizakis, Peter C. von der Ohe, Rémy Beaudouin, Saskia Finckh, Tobias Schulze, Yves Verhaegen, Paul J. van den Brink

**Affiliations:** 1 Cavanilles Institute of Biodiversity and Evolutionary Biology, University of Valencia, Valencia, Spain; 2 IMDEA Water Institute, Alcala de Henares, Spain; 3 Fraunhofer Institute for Molecular Biology and Applied Ecology (IME), Schmallenberg, Germany; 4 Helmholtz Centre for Environmental Research–UFZ, Leipzig, Germany; 5 Swedish University of Agricultural Sciences (SLU), Uppsala, Sweden; 6 Ineris, Verneuil-en-Halatte, France; 7 Plentzia Marine Station (PiE-UPV/EHU), University of the Basque Country, Plentzia, Spain; 8 Ikerbasque, Basque Foundation for Science, Bilbao, Spain; 9 Technological University Dublin, Radiation and Environmental Science Centre, Dublin, Ireland; 10 Goethe University Frankfurt, Frankfurt am Main, Germany; 11 German Environment Agency (UBA), Dessau-Roßlau, Germany; 12 Department of Environmental Science, Stockholm University, Stockholm, Sweden; 13 Baltic Marine Environment Protection Commission, HELCOM Secretariat, Helsinki, Finland; 14 Norwegian Institute for Water Research (NIVA), Oslo, Norway; 15 RIWA-Rijn, Nieuwegein, The Netherlands; 16 Environmental Institute, Koš, Slovak Republic; 17 iES Landau, Institute for Environmental Sciences, University Kaiserslautern-Landau (RPTU), Landau, Germany; 18 Concawe, Brussels, Belgium; 19 Wageningen Environmental Research, Wageningen, The Netherlands; 20 Aquatic Ecology and Water Quality Management group, Wageningen University, Wageningen, The Netherlands

**Keywords:** Diagnostic risk assessment, Bioassays, Toxic pressure, Environmental monitoring, Chemical mixtures

## Abstract

The risk assessment of chemicals relies on multiple tools to quantify the ecological responses of ecosystems to existing chemical pollution. These tools are broadly categorized into three major groups: toxic pressure assessments, bioassays, and ecological monitoring. Here, we examine the strengths and limitations of these approaches, their current level of implementation for freshwater ecosystems across Europe, and their ability to evaluate the impacts of chemicals under field conditions. Additionally, we analyze the correspondence between results obtained from these tools when applied to a monitoring dataset from German streams. Our evaluation showed that no single tool can perfectly characterize the environmental impacts of chemical mixtures. However, each provides distinct lines of evidence, enabling the identification of chemicals driving ecological risks and the biological endpoints most likely to be affected, with ecological monitoring tools having the potential to show long-term ecosystem impairment. Finally, we propose recommendations to better understand the discrepancies between the outcomes of different methods and explore their potential integration into a unified water quality evaluation framework.

## Introduction

Aquatic ecosystems are exposed to multiple organic and inorganic chemicals, including pesticides, pharmaceuticals, metals, and industrial compounds (Busch et al., 2016; Wilkinson et al., 2022). These chemicals form complex mixtures that can affect aquatic organisms simultaneously and in sequence (Van Gils et al., 2020; Rorije et al. 2022). Such complexity highlights the importance of diagnostic (or retrospective) risk assessments, which aim to identify the impacts of chemicals that are in use or that have already been emitted into the environment, relying on advanced tools to characterize chemical exposure and effects, and supporting regulatory decisions on restricting, phasing out, or banning of substances (Vijver et al. 2017; Rico et al. 2021a). To date, our ability to quantitatively assess the risks of chemicals to aquatic ecosystems remains limited due to several challenges. These include difficulties in measuring every single substance that contributes to the environmental exposome (Scholz et al., 2022), the lack of toxicity data for many substances (Treu et al. 2024), the extrapolation of individual-level effects assessed under laboratory conditions to higher levels of biological organization (Schneeweiss et al., 2023), and accounting for multiple (non-)chemical stressors that collectively influence the structure and function of ecosystems (Rico et al., 2016; Van den Brink et al., 2018).

In Europe, the Water Framework Directive (WFD) incorporates multiple lines of evidence to assess the ecological status of aquatic ecosystems and to try to establish causality between measured sources of ecosystem impairment and ecological effects (EC, 2010), following methods that align to the TRIAD approach. The TRIAD approach, introduced in the 1980s by Long and Chapman (1985) for characterizing risks in contaminated sediments, integrates three lines of evidence: chemical (characterization of contaminant exposure and toxic pressure assessment), (eco)toxicological (use of in vivo bioassays), and ecological (biological indices measured under field conditions). This approach was later expanded to account for site-specific features and distinguish between chemical and non-chemical stressor effects (Chapman, 2000). Since then, the approach has been applied in several studies to identify chemicals and biological components affected in contaminated sites (Wolfram et al. 2012, Wolf et al., 2020; Martinez-Haro et al. 2022; Lee, Khim 2022).

**Fig. 1:**
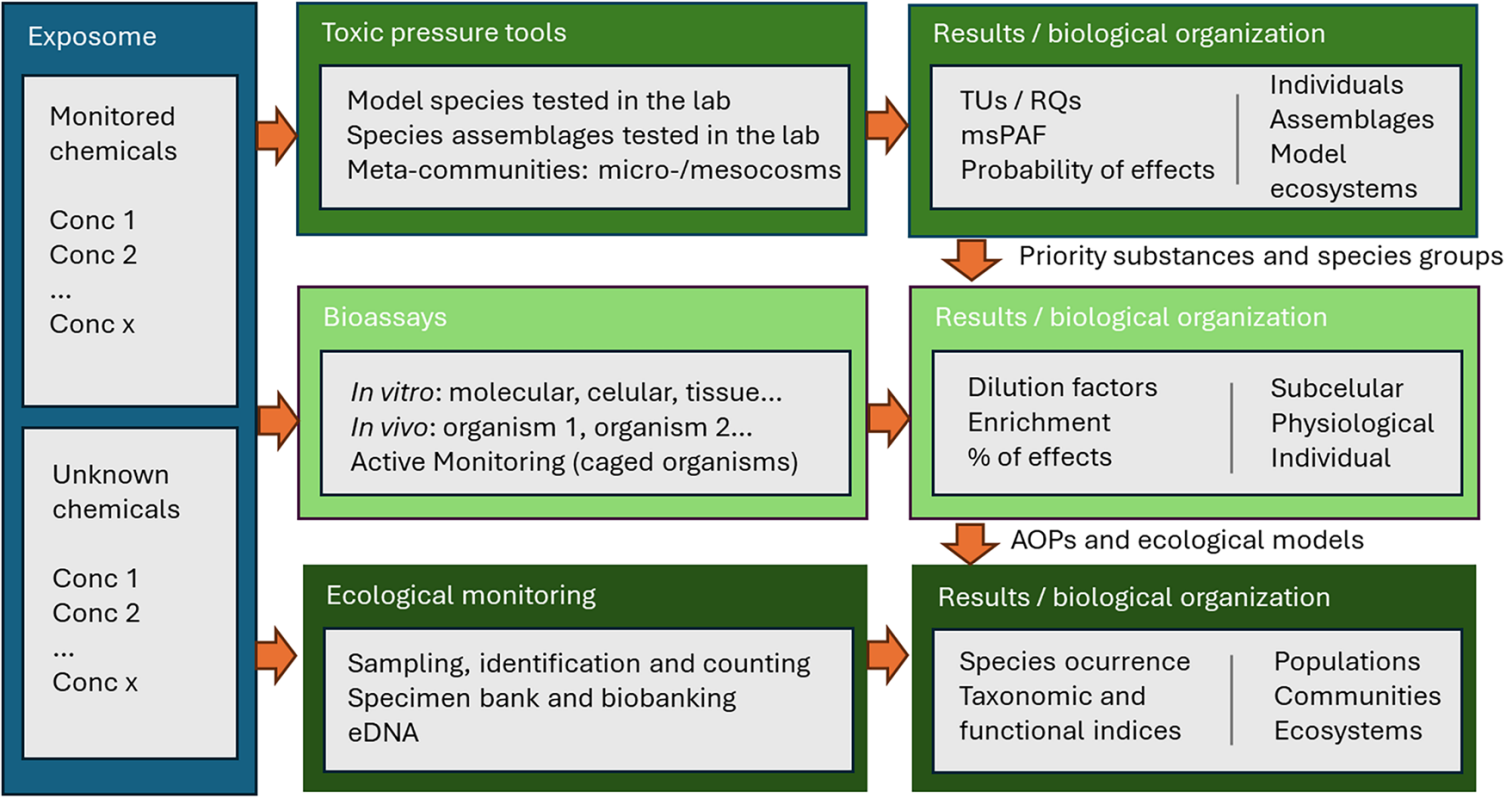
Toolbox for the diagnostic risk assessment of chemicals in freshwater ecosystems

The toolbox that supports the diagnostic risk assessment of chemicals in aquatic ecosystems, in line with the TRIAD approach, has progressed significantly over the past decades (Fig. 1). For instance, toxic pressure assessment metrics, which estimate the likelihood that contaminants will adversely affect biological communities by integrating exposure concentrations with toxicity data, enable the calculation of risk measures based on deterministic or probabilistic methods (De Zwart and Posthuma, 2005; Van den Brink et al., 2002). Traditionally, ecotoxicological assessments were based on in vivo animal tests that provide direct evidence of biological effects in sentinel organisms and biological systems. More recently these have been complemented by in vitro bioassays that range from simple high-throughput screening tools to sophisticated assays using omics techniques. In vitro bioassays allow the characterization of substances’ modes of action, and in combination with fractionation and chemical analysis, even molecular structural identifications and characterization of complex environmental exposures (Escher et al., 2020). Additionally, the repertoire of biological indices that provide quantitative metrics derived from the presence, abundance, and structural and functional parameters of biological communities has grown substantially (Ofogh et al., 2024).

Despite these advances, the complementary use of these tools for diagnostic risk assessment of contaminant mixtures remains limited (Brack et al., 2017; Munthe et al., 2017; Nowell et al. 2024). Integrating these tools into the ecotoxicological risk assessment framework presents opportunities to partially validate the outcomes obtained by other assessment methods, to elucidate toxicological mechanisms, and extrapolate effects to higher levels of biological organization. In light of this, the aims of this paper are: (1) to discuss the development and current implementation status of these tools in diagnostic risk assessment for chemicals in aquatic ecosystems; (2) to evaluate how and to what extent these tools have been employed to validate ecosystem-level effects; and (3) to provide recommendations for further integration of these tools into the ecological risk assessment framework. These discussions are based on the outcomes of a workshop organized by scientists from the NORMAN network (https://www.norman-network.net/), which focuses on improving the risk assessment of chemicals of emerging concern at the ecosystem level. Furthermore, the article presents an evaluation of pesticide mixtures’ effects in German streams, where a suite of diagnostic tools has been used as an example.

## Progress in the development of diagnostic tools

### Toxic pressure assessment tools

The measurement of chemical exposure concentrations under field conditions enables the characterization of theoretical toxic pressure or ecological risks posed by chemical mixtures, utilizing laboratory and/or semi-field toxicity data for individual substances. Laboratory toxicity data for standard test species have been applied to calculate Toxic Units (TUs), which scale measured environmental concentrations of chemicals to a reference toxicity value, such as the acute Effective Concentration for 50% of organisms (EC50) for *Daphnia magna*. Aggregated measures, assuming additivity, have also been developed, such as the summation of TUs for individual compounds in a mixture (Liess and von der Ohe 2005). Additionally, Predicted No Effect Concentrations (PNECs), derived using assessment factors, are employed to calculate risk quotients (RQs) for individual compounds (Ginebreda et al., 2010). One of the most extensive PNEC datasets is maintained by the NORMAN network, encompassing PNECs for 93,556 substances (accessed 9th of March 2025) derived from experimental data and QSAR models for various environmental compartments (https://www.norman-network.com/nds/ecotox/).

The RQ approach is widely recommended for identifying unacceptable ecological risks in regulatory frameworks such as the WFD (EC, 2013). Under the WFD, environmental quality standards are employed using both maximum exposure and annual average concentrations measured across various sites. This approach has been effective in risk prioritization for large chemical monitoring datasets due to its minimal data requirements (e.g., Wilkinson et al., 2022). However, the RQ approach falls short of estimating the actual magnitude of ecological effects on individual trophic levels when the calculated RQ of one or the sum of RQs for multiple chemicals exceeds one.

Laboratory toxicity data for multiple species can be used to construct Species Sensitivity Distributions (SSDs). These distributions allow for a probabilistic estimation of the potentially affected fraction (PAF) of species impacted by individual chemicals. When applied to multiple substances, this approach is referred to as the multi-substance potentially affected fraction (msPAF; De Zwart and Posthuma, 2005). The msPAF is derived by applying SSD parameters to all monitored compounds in a sample, using mixture models such as concentration addition or independent action (De Zwart and Posthuma, 2005). The approach relies on extensive toxicity datasets to estimate a theoretical SSD and the calculation of the proportion of species affected by a contaminant mixture (Posthuma et al., 2019). Similarly to the TU or RQ methods, a significant limitation of the msPAF approach is its inability to quantify the magnitude of impacts on populations and its omission of ecological processes, such as species interactions and indirect effects caused by changes in water quality or ecosystem functions. Despite these limitations, the msPAF approach has been used for diagnostic chemical risk assessments in several countries (Rämö et al., 2018; Posthuma et al., 2019; Rico et al., 2021b; Oginah et al., 2025).

Micro-/mesocosm studies have traditionally been used to derive No Observed Effect Concentrations (NOECs) from which PNEC values are extrapolated for use in high-tier prospective risk assessments (Fig. 1; EC, 2011); EFSA, (2013). The advantage of model ecosystem studies (i.e., micro- and mesocosms) lies in their greater ecological realism, as they account for both direct and indirect chemical effects on populations and community-level responses. Moreover, data from these studies can be used to inform predictive models for ecosystem-level effects. One example is the PERPEST model (Van den Brink et al., 2002), which utilizes a database of model ecosystem experiments to estimate the probability of slight or severe effects on various biological endpoints caused by chemical mixtures measured in the environment. This is achieved using machine learning tools such as case-based reasoning. Currently, the source dataset is populated with model ecosystem studies focused on pesticides, which means that, to date, the model has been used exclusively for risk assessments of these compounds (Van den Brink et al., 2006a; Rämö et al., 2018). However, the use of probabilistic approaches based on micro- and mesocosm experimental results, or similar high-tier datasets, shows significant promise for advancing diagnostic chemical risk assessments encompassing higher levels of biological organization (e.g., Mentzel et al., 2024).

### Bioassays

A comprehensive battery of bioassays is available to evaluate the impact of chemical mixtures at the sub-individual and individual levels (Fig. 1; Schuijt et al., 2021). These bioassays, which include in vitro and in vivo techniques, enable the detection of chemical effects on morphological and physiological responses in model species and/or (sub)cellular systems (Barata et al., 2007; König et al., 2017). In vitro bioassays utilize cell cultures or subcellular systems derived from organisms such as mammals or fish, or modified bacteria. Reporter gene assays are particularly popular, as they amplify and visualize cellular processes such as the activation of estrogen receptors by estrogenic chemicals. When native nuclear receptors or transcription factors—activated at specific steps in cellular toxicity pathways—bind to chemicals, they trigger the transcription and translation of a reporter gene. This gene encodes easily measurable proteins, such as fluorescent proteins or enzymes (e.g., β-galactosidase or luciferase). The resulting measurable response (e.g., fluorescence intensity or enzyme activity) correlates with the degree of receptor binding, providing a direct proportional measure of the chemical’s (toxic) effect, which is usually expressed in dilution times needed to find a non-toxic effect (i.e., a non-significant measurable response of the endpoint or the measured enzymatic activity; Escher et al., 2021). Some of these bioassays have been used to establish effect-based trigger (EBT) values for surface water (Escher et al., 2018; Neale et al. 2023). Furthermore, these assays can be supplemented with sequential chemical analyses to confirm the specific chemicals responsible for the observed biological activity. Additionally, fractionation techniques, applied under the framework of Effect-Directed Analysis (EDA), can be used to select chemical compounds exhibiting specific activities from complex contaminant mixtures (Brack et al., 2016), helping in the risk prioritization of substances and on the determination of toxic mode of action of single substances or mixtures.

Active monitoring is conducted using sentinel organisms either caged within the study area (Liess and Schulz 1999) or tested under laboratory conditions using water samples collected from the field, which are commonly termed in vivo bioassays. As contaminants often occur at low concentrations without obvious effects, water samples used for in vivo testing are typically enriched, e.g., via solid-phase extraction, to cover a wider range of exposure concentration levels and amplify response detection (Neale et al., 2018). The assays measure a broad spectrum of response endpoints, encompassing both exposure biomarkers that identify pollutants through tissue bioaccumulation analysis or metabolite detection, and effect biomarkers that evaluate biological responses to pollution. Effect biomarkers include molecular and biochemical responses such as oxidative stress, acetylcholinesterase inhibition, and DNA damage, as well as cellular and histopathological alterations like lysosomal membrane stability, hepatic lesions, and micronucleus formation. Additionally, physiological and behavioral changes including reproductive output, swimming behavior, and heart rate are assessed, along with population-level indicators such as sex ratio. These assays frequently employ model organisms like zebrafish (*Danio rerio*) and water fleas (*Daphnia magna*) for laboratory testing and *Gammarus sp*., *Dreissena sp*., or sticklebacks for field monitoring, with results typically expressed as percentage effect relative to acute endpoints (for further details see Schuijt et al., 2021).

One of the primary strengths of both in vitro and in vivo techniques is their capacity to identify key toxicity mechanisms influenced by chemicals in environmental samples. When conducted with water extracts, these techniques primarily capture the effects of retained organic chemicals. Conversely, when water is directly dosed, they can reflect the combined impacts of organic contaminants, inorganic contaminants, and organic matter, as well as the influence of the sample’s physico-chemical properties (e.g., pH or dissolved oxygen levels), which may contribute to the mixture’s overall toxicity. These techniques are highly effective for detecting physiological damage at the cellular or individual level in sentinel organisms, providing valuable insights into cumulative stress responses. However, their ability to predict broader population- or ecosystem-level impacts remains limited.

### Ecological monitoring tools

Ecological monitoring typically involves collecting biological samples to characterize aquatic ecosystems’ ecological status over time. This approach enables assessment of responses to both chemical and non-chemical stressors, which can be jointly monitored (Fig. 1). Biological samples are used to calculate integrated ecological indices that account for ecosystems disturbances at various trophic levels, e.g., primary producers, primary and secondary consumers, or predators. These indices are integral components of large-scale monitoring programs, serving to assess the ecological status of water bodies as part of the European WFD (Birk et al. 2012). Diverse metrics have been established in many of the EU member states, whose results have been compared in laborious intercalibration studies to determine reference conditions and ecological status classes for different water body types (Furse et al. 2009). These include, for example, the more general taxonomic indices for macroinvertebrates, such as diversity, richness or evenness, and the Saprobic Index (Rolauffs et al., 2004), the % of Ephemeroptera, Plecoptera, Trichoptera (%EPT) index (Weber, 1973) or the Biological Monitoring Working Party (BMWP) index (Hawkes 1997), which indicate general degradation compared to a reference situation. One of the main advantages of these methods is that they attain a high level of ecological realism, thus providing an integrated measure of community differences with undisturbed systems due to natural or anthropogenic perturbations. Moreover, such indices can be harmonized into Ecological Quality Ratios, which are used for European-level assessments of aquatic ecosystems (Solheim et al. 2025). However, the use of these ecological indices for the diagnostic risk assessment of potentially toxic chemicals is challenging, mainly due to their correlation with a wide range of non-chemical stressors, including organic matter pollution, nutrients, habitat modification or hydrological alterations (Schuwirth et al. 2015; Rico et al. 2016; Liess et al. 2021). This correlation has led to discussion on whether chemical-specific indices (i.e., indices that unequivocally determine the effects caused by chemicals with specific toxic mode of action) could be developed, with limited success so far (Schuwirth et al. 2015).

During the last decade, there has been a plea for the development of ecological indices based on biological traits, as these allow clearer links to be established between chemical effects and functional ecosystem responses, facilitating comparison of community responses across water bodies and (eco)regions (Menezes et al. 2010; Aazami et al. 2015). Several trait-based approaches exist, such as those for insecticides (Rico and Van den Brink, 2015) and salinity (Schäfer et al., 2011). Among these, the SPEAR index is notable for incorporating species’ relative sensitivity to pesticides and specific traits related to the probability of exposure and recolonize/recover from contaminant pulses (Liess and von der Ohe, 2005; Von der Ohe and Goedkoop 2013). The SPEAR index has been used in several research studies in Europe and other continents (see Hunt et al. 2017 and references therein) to evaluate the effects of pesticides, but the widespread inclusion of such trait-based indices into regulatory risk assessment has been limited so far.

Ecological monitoring research has also evolved to incorporate molecular tools, such as environmental DNA (eDNA) metabarcoding techniques, to estimate species diversity and assess chemical impacts on ecosystem functions (Zhang, 2019). A key advantage of these methods is their non-lethal nature for certain monitored organisms, minimizing habitat disruption while enabling assessments of diversity across a wide taxonomic spectrum, including rare species often overlooked by conventional sampling methods (Deiner et al., 2016). Over the past decade, there has been a growing effort to adapt indices used in the WFD to incorporate eDNA monitoring data for fish and other biological quality elements (Hering et al., 2018; Pont et al., 2021). However, the use of eDNA monitoring to assess chemical risks—particularly to establish connections between exposure and adverse effects across different levels of biodiversity—remains relatively limited (Schuijt et al., 2024). Moreover, linking chemical exposure to population- or community-level effects using eDNA data requires robust statistical correlations or the development of calculated indices.

The establishment of environmental specimen banks and biobanking programs (based on gene diversity studies) represent a valuable approach to collecting high-quality biological samples in a standardized manner through long-term ecological monitoring initiatives (Garmendia et al., 2015). Cryopreserved specimen banks facilitate spatial and temporal comparisons between historical and present samples with chemical datasets, providing insights into the historical evolution of pollutant effects or abrupt (genetic) diversity changes caused by the environmental release or the ban of certain contaminant classes. Ideally, these efforts require a thorough evaluation of the chemical fingerprint at the sites where the specimens and biobanking samples are collected or, at the very least, robust knowledge of nearby contamination sources. To date, specimen banks have been successfully implemented in countries like Germany to monitor changes in chemical bioaccumulation patterns across various aquatic organisms and pollutants (e.g., https://www.umweltprobenbank.de/en/). Meanwhile, biobanking has primarily been applied within conservation biology. The integration of data from both specimen banks and biobanking, alongside other risk assessment tools, holds significant promise for inferring long-term chemical effects on aquatic biodiversity and environmental health.

## Performance evaluation of diagnostic tools

The tools outlined above have been utilized to identify chemicals of concern and ecosystem components particularly susceptible to damage (in a general sense) based on measured chemical exposure levels. Despite their inherent differences, these tools are expected to yield complementary results, highlighting both the chemicals and biological components at risk. Several studies have attempted to compare the outcomes produced by different diagnostic tools and to identify potential sources of discrepancies through evaluation exercises (hereafter referred to as evaluations or evaluation studies).

Such evaluations have often focused on comparing toxic pressure tools and ecological monitoring outcomes. For instance, field monitoring data has been employed to correlate TUs with various taxonomic indices, such as the BMWP, the Saprobic Index, and the %EPT. Significant negative correlations were observed, though the R^2^ values were generally modest, typically not exceeding 0.3 in large rivers like the Danube (e.g., Rico et al., 2016), and values of 0.4 (Liess et al., 2021) and 0.7 (Liess and von der Ohe 2005) in streams and small rivers. Moreover, the sum of pesticide TUs was found to correlate with species richness derived from rarefaction curves, suggesting that current pesticide mixtures may be responsible for up to a 42% decline in species richness (Beketov et al., 2013). Toxic pressure units have also been extensively assessed against trait-based indices, particularly the SPEAR_pesticides_ index, showing significant correlations across various water bodies (Beketov et al., 2013; Liess et al., 2021). Schäfer et al. (2013) evaluated the predictive capacity of four toxic pressure units (TUs based on *Daphnia magna*, TUs for the most sensitive species, TUs based on SSD HC5, and the msPAF approach) against the SPEAR_pesticides_ index using samples from five European countries. Their findings indicated that that either TUs based on HC5 values (in Australia), TUs based on the most sensitive species (Denmark, France and Germany), or the msPAF values (Spain) performed best, depending on the location.

PAF and msPAF calculations have also been compared with field-measured changes in species richness and other biological indices. Carafa et al. (2011) reported significant negative correlations between msPAF values and biotic indices for macroinvertebrates (BMWP) and diatoms (IPS) in Spanish rivers. However, other studies investigating similar relationships with fish richness demonstrated weaker correspondence, likely due to confounding factors such as river flow connectivity and predominant non-chemical stressors (Posthuma and de Zwart, 2006). Posthuma and de Zwart (2012) investigated the relationship between msPAF-EC50 values (calculated for 45 compounds using laboratory acute EC50 data) and macroinvertebrate abundance data obtained from ditches and streams in the Netherlands. Their study involved modeling population abundance declines for each taxon relative to reference conditions, which are often unavailable or influenced by multiple abiotic variables. Using Generalized Linear Models, they predicted population abundance under both chemical stress and non-chemical stress conditions. Their findings showed that the fraction of taxa exhibiting a 50% or greater abundance reduction corresponded well with msPAF-EC50 predictions for the most sensitive species. However, msPAF tended to underestimate population declines for less sensitive taxa, possibly due to species interactions. Additionally, some taxa exhibited population increases, which were not predicted by the msPAF approach, being identified as a major source of discrepancy between both methods. Posthuma et al. (2020) identified a strong correlation between msPAF-EC50 values (95th percentile) for 24 priority substances monitored in European rivers and their ecological status. They proposed msPAF-EC50 thresholds for assessing the ecological status of rivers, in line with the classification system established by the Water Framework Directive (WFD). More recently, Oginah et al. (2025) quantified toxic pressure levels at over 1,000 sites in the Netherlands using the msPAF approach and compared these values to macroinvertebrate abundance and species richness loss. Their findings revealed that macroinvertebrate abundance and richness generally decrease with increasing toxic pressure, with a nearly 1:1 relationship observed between msPAF-calculated values and the potentially disappeared fraction of species.

TUs and msPAF results have also been compared with outcomes from micro- and mesocosm experiments. TUs calculated with toxicity data for standard test species show strong correlations with observed effects in model ecosystem experiments involving pesticides (Brock et al., 2000a, 2000b). Furthermore, data from these experiments have been used to assess the protectiveness of the Hazard Concentration 5% (HC5) values derived from SSDs built with laboratory toxicity data. In most cases, these HC5 values were between 1.1 and 4 times lower than the lowest population NOEC obtained from micro-/mesocosm experiments (Maltby et al., 2005, 2009; Van den Brink et al., 2006b; Del Signore et al., 2016). Studies validating the SSD approach with model ecosystem experiments have generally focused on threshold (HC5) values rather than the entire SSD curve (Hose and Van den Brink, 2004). Rico et al. (2018) compared SSDs built with chronic laboratory toxicity data for the insecticide imidacloprid and a mixture of five neonicotinoid insecticides with the fraction of species significantly affected in a mesocosm experiment. They found a very good agreement between the predictions made with the PAF and msPAF values obtained from the SSDs and the observed effects in the mesocosm experiment at different toxic stress levels, supporting the use of such predictive tools for determining direct toxic effects in (semi-)natural species assemblages.

Models like the PERPEST model, which leverage data from micro- and mesocosm experiments, enable the calculation of chemical (or mixture) effects on structural and functional ecosystem parameters. However, comparisons between PERPEST predictions and other toxic pressure tools, such as TUs or msPAF indices, remain unexplored. Such comparisons would be highly valuable, as toxic pressure tools primarily focus on direct chemical effects, whereas the PERPEST model accounts for both direct and indirect effects. Moreover, these approaches rely on fundamentally different datasets—laboratory toxicity tests for toxic pressure tools versus outdoor mesocosm experiments for the PERPEST model. Consequently, any observed alignment between the two would significantly bolster confidence in diagnostic assessments and improve their reliability.

The integration of in vivo and in vitro bioassay monitoring data with ecological monitoring data or toxic pressure metrics remains a relatively unexplored field. In vitro assay results are often compared directly with EBT values, which are typically derived from environmental quality standards by incorporating relative potency estimates and mixture effects (Escher et al., 2018; Neale et al. 2023). Within this framework, in vitro bioassays are frequently regarded as analytical tools, used to identify complex chemical mixtures and link them to chemical analytical results through the application of mixture models. For certain endpoints, such as hormone-mimicking effects measured using estrogenicity assays, nearly 100% of the observed mixture effects can be attributed to detected estrogenic chemicals (Könemann et al., 2018). Similarly, photosynthesis inhibition is largely driven by known herbicides (Glauch and Escher, 2020). However, the explanatory power diminishes significantly for less specific toxicity pathways that are further downstream from molecular initiating events. Despite the detection of dozens of chemicals in surface water or environmental samples, the modeled and measured mixture effects of these detected chemicals often explain less than 1% of the observed effects for such endpoints (Neale et al., 2020). This apparent discrepancy may initially seem surprising but becomes more understandable when considering that a typical non-target chromatogram contains tens of thousands of peaks, many of which represent unknown chemicals likely contributing to the observed mixture effects (Escher et al., 2020). A recent study investigating the presence of 225 chemicals in Dutch surface waters found that in vitro bioassay results could be directly linked to detected chemicals in only 6% of cases. Furthermore, the detected chemicals accounted for just 1–17% of the observed effects, suggesting that the majority of the effects were likely caused by undetected chemicals (Boonstra et al., 2025).

## Multiple diagnostic tools applied to German streams

This section demonstrates how various diagnostic tools can be utilized to assess the ecological risks posed by realistic contaminant mixtures. The analysis is based on a dataset that includes invertebrate monitoring data, in vitro bioassay results, and pesticide concentrations measured in German streams (obtained from Neale et al., 2020; Liess et al., 2021). The subsequent sub-sections provide a detailed description of the dataset, outline the diagnostic indicators calculated, and explain the methods employed to enable a comparative assessment of different diagnostic tools.

### The dataset

The dataset encompasses 56 sampling sites randomly selected from those monitored by Liess et al. (2021). Sampling was conducted between April and June in 2018 and 2019, using event-driven sampling (i.e., automatic sampling triggered by runoff events), which showed a higher number of substances as compared to samples taken during fair-weather conditions (Neale et al., 2020). Sites with certain wastewater treatment plant (WWTP) influence were excluded, to primarily focus on effects caused by pesticides and exclude potential indirect effects caused by ammonia and other organic decomposition by-products. Pesticide and metabolite analysis involved 108 substances, detected using UHPLC-MS/MS with detection limits in the nanograms per liter (ng/L) range. Macroinvertebrate monitoring was conducted during June of 2018 or 2019 using the multi habitat sampling method. Macroinvertebrate samples were identified to the lowest taxonomic resolution possible. Detailed methodologies for pesticide and macroinvertebrate sampling and analysis can be found in Liess et al. (2021). Water samples were also used for in vitro bioassays targeting endpoints such as cytotoxicity, estrogen receptor activation (ERα), aryl hydrocarbon receptor activation (AhR), peroxisome proliferator-activated receptor activation (PPARγ), and oxidative stress response (AREc32). The methods used for these in vitro tests are described in detail in Neale et al. (2020).

### Data analysis

The following diagnostic tools and indices were calculated from the dataset:

#### Toxic pressure assessment

The maximum pesticide concentrations measured at each sampling site were used by Liess et al. (2021) to calculate the log(TUmax) (hereafter TUmax) based on LC50 toxicity values for *Daphnia magna* or *Chironomus sp*. Furthermore, these maximum pesticide concentrations were used to calculate acute and chronic msPAF values for aquatic organisms, following the SSD parameters and calculation methods (assuming concentration addition) outlined by Posthuma et al. (2019), and implemented in the Key Factor Toxicity tool (STOWA, 2025). Additionally, the probability of observing significant effects on macroinvertebrates based on the pesticide concentrations at the sampling sites was calculated using the PERPEST model. Effects were defined as the sum of the probabilities of observing effect classes 2 and 3. These correspond to slight effects (partial reductions in population abundance on individual sampling days) and clear effects (severe reductions in population abundance over several consecutive sampling days), as documented in the micro- and mesocosm experiments included in the PERPEST database (Van den Brink et al., 2002). The PERPEST model calculations were performed for pesticides with a maximum hazard unit (the ratio of the maximum measured concentration to the hazardous concentration for 50% of organisms (HC50) obtained from the compound´s SSD) exceeding 0.002, while the rest were excluded from the PERPEST calculations as were assumed not to contribute to the calculated toxic pressure. The 0.002 threshold was derived by applying two extrapolation factors: A factor of 5 to extrapolate from HC50 to HC01 (based on the commonly reported SSD slope of 0.7 by Posthuma et al., 2019), and a factor of 100 to account for the additive toxic pressure from the approximate 100 chemicals included in the analysis.

#### In vitro assessment

The effects detected with four reporter gene assays were expressed in effect concentrations triggering 10% of maximum effect for the activation of the arylhydrocarbon receptor (AhR), the estrogen receptor (ERα), the peroxisome-activated receptor (PPARy) and the activation of the oxidative stress response 50% over the control ECIR1.5 (AREc32). The cytotoxicity IC10 (i.e., inhibitory concentration 10%) measured for each of the four in vitro bioassays (AhR, Erα, PPARy and AREc32) by Neale et al. (2020) in the water samples from each site was transformed into in vitro TUs (TU = 1/IC10). Subsequently, the average of the four in vitro TUs was calculated and used here as an integrated measure of in vitro cytotoxicity.

#### Ecological monitoring

The macroinvertebrate measurements (individuals/m^2^) were used here to calculate four common biological indices: total abundance, species richness, diversity (Shannon), and evenness, using the CANOCO software version 5 (ter Braak and Šmilauer 2012). Furthermore, SPEAR values were obtained from the calculations done by Liess et al. (2021).

Finally, a Pearson correlation analysis was done to assess the relationship between the toxic pressure tools employed here (TUmax, msPAF acute, msPAF chronic and the probability of finding effects as calculated with PERPEST), the in vitro cytotoxicity indicator (in vitro TUs) and the ecological monitoring indicators (abundance, richness, diversity, evenness and the SPEAR index). The Pearson correlation analysis and graphical interpretation was done with the SR plot software (Tang et al. 2023). The correspondence between the different tools and indicators was evaluated based on the significance of the Pearson correlation coefficients. Significant correlations were plotted using linear regression models built with Microsoft EXCEL (Microsoft Corporation 2024). The pesticide and invertebrate monitoring data used in this study, together with the in vitro test outcomes and the calculated indices are provided in the Supporting Information file.

### Study outcomes

**Fig. 2:**
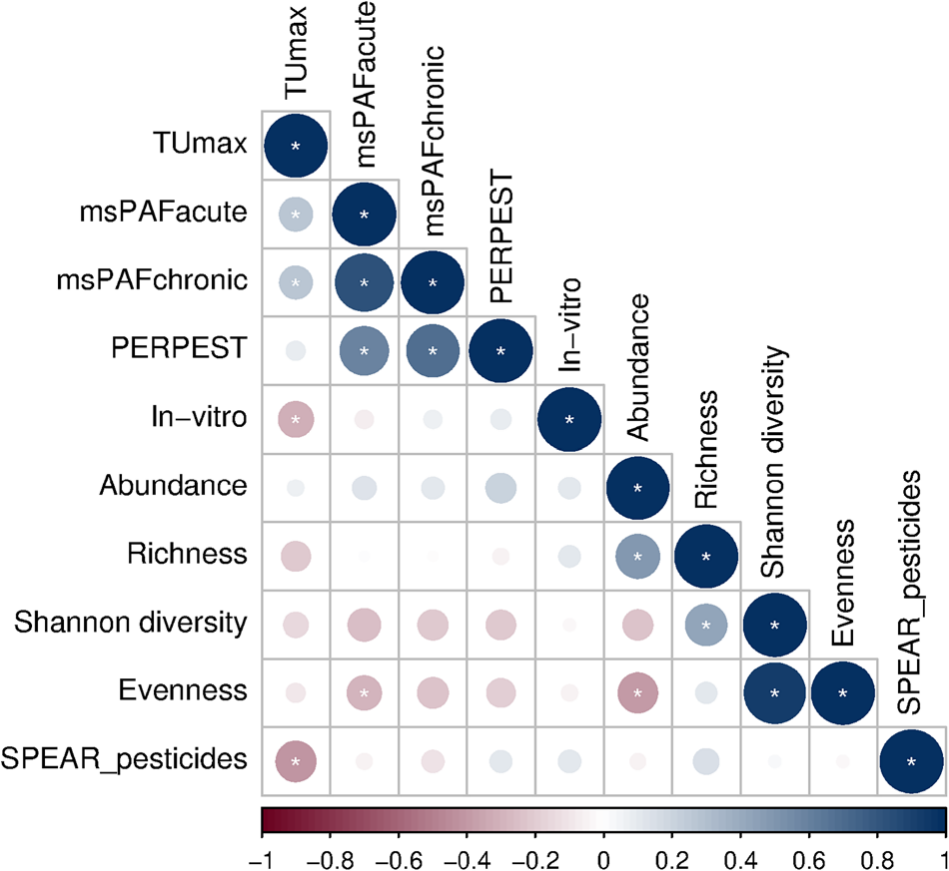
Pearson correlations between toxic pressure (i.e., log(TUmax), msPAFacute, msPAFchronic, PERPEST probability of effects), in vitro toxicity (i.e., in vitro TUs, expressed as 1/IC10 values) and ecological monitoring indices (i.e., abundance, richness, Shannon diversity, evenness, SPEARpesticides) calculated for pesticides and macroinvertebrates monitored in German streams based on the data provided by Liess et al. (2021) and Neale et al. (2020). The circles show the size and sign of the Pearson correlation coefficients (blue = positive, red = negative). Significant correlations (*p*-value < 0.05) are marked with a star inside of the circle. The dataset used for the Pearson correlation analysis is provided in the Supporting Information file

**Fig. 3:**
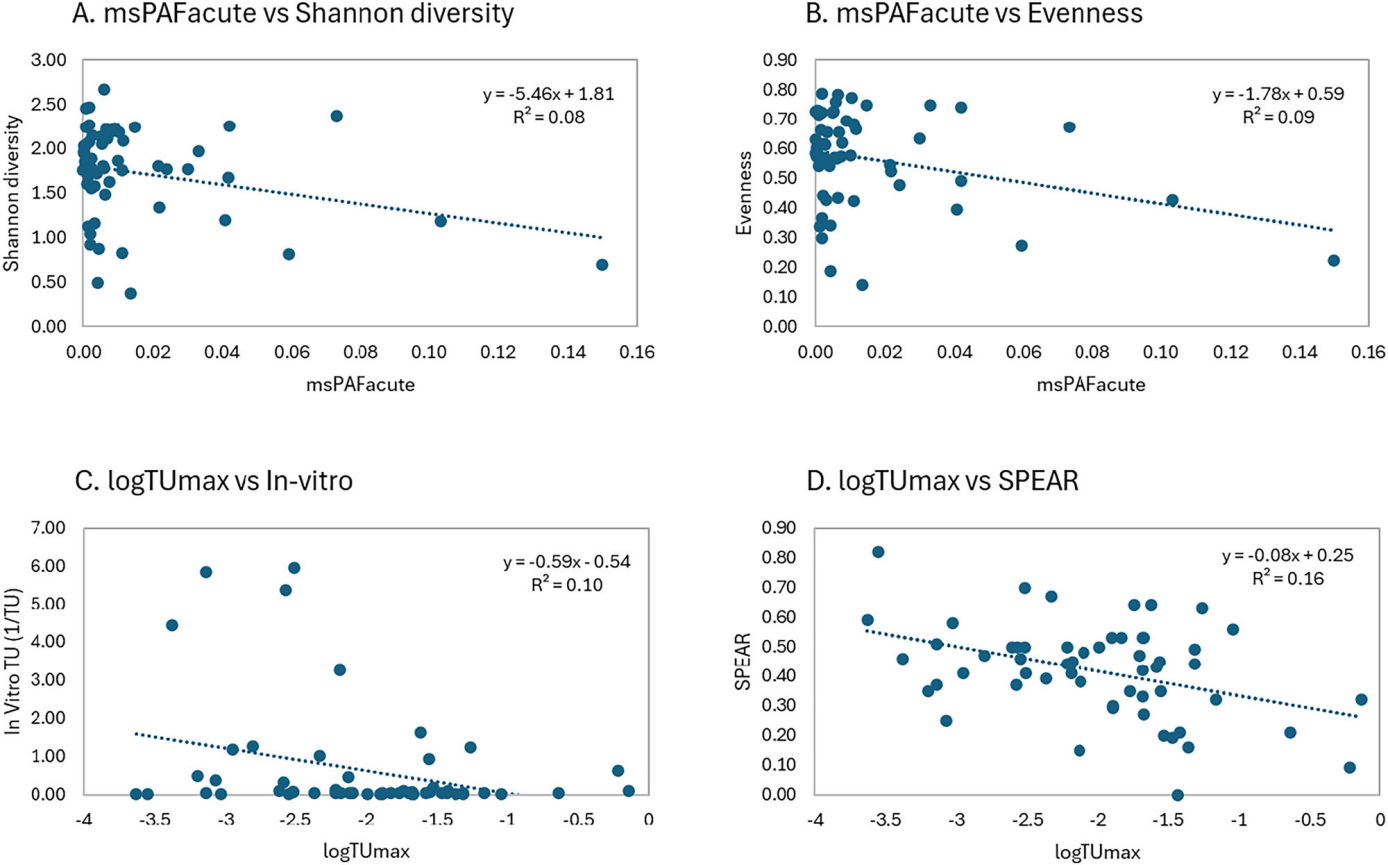
Linear regression models showing the relationship between (**A**) the msPAFacute and the Shannon diversity index, (**B**) the msPAFacute and the Evenness index, (**C**) the logTUmax and the in vitro TU, and (**D**) the logTUmax and the SPEAR index

Figure 2 presents the Pearson correlations between the various toxic pressure metrics, in vitro bioassay results, and ecological monitoring indices. Significant correlations were detected between the TUmax and other indicators. The strongest was a negative relationship with the SPEARpesticides index (Pearson r = −0.40; *p* = 0.002; R² = 0.16;), indicating that higher TUmax values correspond to lower abundance of pollution-sensitive invertebrate species. TUmax also positively correlated with msPAF acute and chronic (Pearson r = 0.28 and 0.29; *p* = 0.04; R² = 0.08 for both), implying that elevated TUmax increases the percentage of potentially affected species calculated with SSDs. Interestingly, TUmax showed a negative correlation with in vitro TUs (Pearson r = −0.31; *p* = 0.02; R² = 0.10), suggesting that higher toxic pressure in standard macroinvertebrate tests aligns with greater stress in bioassays (note that in vitro TUs represent 1/IC10 values; Fig. 3). Among the in vitro results, ERα activation was notably high, alongside markers of untreated wastewater contamination. This points to inputs from human waste—likely via road runoff or discharges from small urban areas with limited sewage treatment—despite the streams being selected for agricultural influence. Other bioassay endpoints showed activation, though mixture effects generally remained below established effect-based thresholds (EBTs). TUmax was not significantly correlated with trait independent measures of the invertebrate community structure.

A significant positive correlation was observed between msPAF (acute and chronic) and the probability of macroinvertebrate effects predicted by the PERPEST model (Pearson r = 0.58 and 0.68, respectively; *p* < 0.001; R² = 0.34 and 0.46). This relationship may stem from the PERPEST model’s reliance on HC50 values derived from SSDs for the calculation of pesticide hazard units for querying its micro-/mesocosm database. Additionally, msPAF acute exhibited a significant negative correlation with species evenness (Pearson r = −0.28; *p* = 0.03; R² = 0.09;), suggesting that exceeding the 5% species protection threshold (msPAF acute) reduces the prevalence of rare and potentially sensitive taxa (Fig. 3).

The results of this case study demonstrate that, among the toxic pressure indicators evaluated, the TU approach performs best, as it effectively predicts both bioassay outcomes and biological monitoring indicators such as SPEARpesticides. However, the overall correlation coefficients were relatively low, suggesting that confounding factors influence each of these tools, limiting their ability to perfectly align. Additionally, none of the indicators showed a statistically significant correlation with classical invertebrate biodiversity metrics, such as the Shannon index and taxonomic richness. Nevertheless, while the correlations were not significant, the msPAF approach (acute and chronic) and the PERPEST model exhibited the strongest predictive capacity among the methods tested on such biodiversity indices (Figs. 2 and 3).

## Uncertainties and recommendations to improve the diagnostic framework

**Fig. 4:**
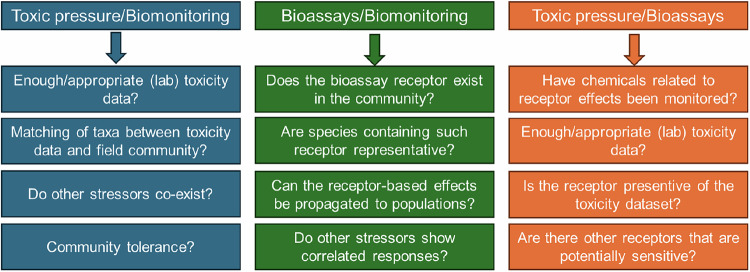
Standing questions pinpointing to the potential misalignments between the results obtained from various diagnostic tools used in chemical risk assessment

The evaluation studies (discussed above) and the findings of the case study on German streams highlight significant data gaps and inconsistencies in the implementation and correlation between different diagnostic tools, as summarized in Fig. 4. This section outlines key data gaps and methodological advancements necessary for achieving better alignment between the results of different diagnostic tools.

### Toxic pressure tools vs biomonitoring

Discrepancies between toxic pressure assessments and biomonitoring outcomes often arise from inadequate toxicity data to represent the species present in the ecosystem under investigation. This limitation frequently stems from the reliance on a small set of standard test species for TU calculations or from the lack of toxicity data required to construct SSDs. Promising advancements include the use of machine learning algorithms that leverage chemical and biological predictors (e.g., taxonomy, physiology, or traits) to estimate the sensitivity of untested (non-standard test) species (Zubrod et al., 2024) and to expand species coverage in SSDs (Liang et al., 2024), thereby improving the robustness of toxic pressure assessments.

Another limitation of toxic pressure assessments is their focus on the measurable fraction of the exposome, excluding numerous unknown chemical compounds (Boonstra et al., 2025). For instance, a nationwide study in Germany assessed 464 pesticide compounds and found that additive mixture effects increased aquatic risk indicators (e.g., for invertebrates and algae) by 3.2 times under realistic worst-case scenarios compared to single-pesticide predictions (Weisner et al., 2021). These findings emphasize the importance of incorporating as many chemicals as possible in toxic pressure calculations and potentially investigating the need for a Mixture Assessment Factor (Backhaus, 2023), as done in prospective risk assessment, when chemical data are limited or unrepresentative of the full exposome. The Mixture Assessment Factor was designed for managing risks from unknown unintentional mixtures in prospective risk assessment, but an adapted version of it could be used when only a limited number of substances is measured in diagnostic risk assessments.

Toxic pressure assessments typically rely on concentrations from single grab water samples, while biomonitoring outcomes often reflect cumulative exposures over extended periods and across multiple exposure matrices, such as sediments and biota (Ijzerman et al., 2024). To address this, toxic pressure calculations should incorporate data from integrative multi-species, multi-compartment approaches (Miller et al., 2021; Manjarrés-López et al., 2025) using passive sampling devices (Shaw and Mueller, 2009). This could provide a more realistic estimation of long-term toxic pressure on aquatic populations and communities.

Furthermore, toxic pressure assessments predominantly focus on chemical stressors, yet aquatic communities are simultaneously exposed to a range of non-chemical stressors, including altered water flow, salinity, temperature extremes, or biological invasions (Birk et al., 2020). While the integration of non-chemical stressors into toxic pressure assessments remains limited, progress has been made. For example, metal toxicity data can be adjusted for salinity and pH gradients to calculate TUs, and temperature-dependent SSDs could be derived for some chemicals, either based on experimental data (Wang et al., 2019) or extrapolated toxicity data using temperature-dependent toxicokinetic-toxicodynamic models (Mangold-Döring et al. 2022).

Environmental toxicants, such as pesticides, impose strong selective pressures on various species. While the evolution of pesticide resistance in agricultural fields is well-documented, evidence of adaptation in non-target species exposed to these toxicants remains inconsistent (Bundschuh et al., 2023). Some studies suggest that species diversity plays a critical role in the development of toxicant tolerance by modulating the balance between intra- and interspecific competition (Becker and Liess, 2017). Multispecies models offer a promising complementary approach for quantifying the net effects of interspecific interactions on ecosystem responses to chemical stress, but initial efforts reveal their limited capacity to replicate community-level responses observed under (semi-)field conditions (Loerracher et al., 2023). Future research should focus on enhancing the ecological realism of model predictions by incorporating factors such as microevolutionary adaptation, internal and external population recovery pathways, and species’ adaptive behaviors, which collectively enhance community resilience to chemical pollution.

### Bioassays vs biomonitoring

The limited alignment between bioassay results and ecological monitoring outcomes can be attributed to several factors. A primary challenge lies in extrapolating in vitro effects to in vivo outcomes, which stems from multiple issues. First, chemical bioavailability and toxicokinetics in in vitro bioassays often fail to account for delayed or cumulative effects, limiting their ability to predict certain apical responses (Yoon et al., 2012; Grech et al., 2019). Second, there is a scarcity of in vitro bioassays that adequately represent the diversity of aquatic organisms. Many currently available assays were developed for human toxicology and focus on conserved vertebrate receptors, such as those found in fish, while largely neglecting other taxa like invertebrates and primary producers. Third, the in vitro-to-in vivo extrapolation models are primarily designed for human toxicology, creating significant gaps when applied to aquatic organisms (Villeneuve et al., 2019; Brinkmann et al., 2016; Wang et al., 2022). To address these limitations, research efforts should focus on expanding the in vitro toxicity testing battery to include a broader range of receptors characteristic of freshwater ecosystems (beyond vertebrate taxa). Additionally, improved methods for extrapolating in vitro responses to in vivo apical effects are needed.

Moreover, our understanding of how in vitro results translate to population and community-level outcomes remains limited. The Adverse Outcome Pathway (AOP) framework provides a valuable approach for describing how chemical exposure and in vitro effects propagate across different levels of biological organization (Ankley et al., 2024). In the past decade, significant progress has been made in describing molecular initiating events and key events and linking them to ecologically relevant endpoints such as survival, growth, and reproduction (e.g. Baldwin et al., 2009). However, AOPs have predominantly focused on model organisms, limiting their applicability across a broader range of species. There is a pressing need to develop knowledgebases and computational tools to enhance inter-chemical and inter-taxa extrapolation and to support quantitative predictions of adverse effects at higher levels of biological organization (Kramer et al., 2011; Ankley et al., 2024).

Another complicating factor is the potential for correlated responses of in vitro results to other stressors present in environmental samples. Field studies have revealed significant natural variability in the enzymatic profiles of aquatic organisms (e.g., acetylcholinesterase, catalase, glutathione-S-transferase) driven by environmental factors such as temperature, salinity, and other physicochemical variables (Menezes et al., 2006; Pfeifer et al., 2005; Ippolito et al., 2017). Understanding this variability is essential for disentangling chemical-induced effects from those arising due to (natural) environmental influences.

### Toxic pressure vs bioassays

Discrepancies between toxic pressure assessments and bioassay results may stem from the presence of undetected toxic chemicals in environmental samples, which are excluded from toxic pressure calculations due to analytical limitations. In such cases, employing EDA techniques to reduce sample complexity through fractionation can aid in identifying toxicologically relevant compounds (Escher et al., 2020). Furthermore, combining suspect screening and non-target screening approaches allows for the identification of compounds without the need for reference standards, thereby broadening the scope of chemical analysis (Hollender et al., 2023).

Another critical factor is the appropriate selection of species and toxicological endpoints for toxic pressure calculations. Bioassays produce results that are specific to certain species or taxonomic groups; therefore, toxic pressure assessments must align with these parameters to enable accurate comparisons. For instance, bioassay results derived from fish or mammalian cell lines may not correlate well with toxic units TUs or msPAF calculated from datasets that include aquatic invertebrates or plants. This misalignment likely contributed to the weak correlation observed in the German streams case study.

Even when taxonomic alignment is achieved, false negatives may still occur if the receptor specificity of the bioassay does not encompass the broader range of receptors present in the model species. As discussed earlier, addressing these gaps requires the integration of additional receptors through alternative bioassays, enhancing the robustness and reliability of toxic pressure evaluations.

### Towards an integrative framework

Each group of diagnostic tools described above may be represented by different (groups of) indices, constituting multiple criteria to assess chemical impacts on ecosystems. The integration of these results to provide robust risk conclusions and to define management decisions has been debated under the multiple Lines of Evidence (LOEs) approach (US EPA, 1998; EFSA, 2021). This approach assumes that combining multiple independent lines reduces uncertainty and increases confidence in risk estimates, so that combinations of such lines of evidence are required to achieve a robust conclusion. The Weight-of-Evidence (WoE) approach has been dedicated to the integration of multiple LOEs based on qualitative methods, grounded on comparative tables and triangulation methods, or quantitative methods, using matrix-based approaches and Bayesian networks (Weed 2005; Linkov et al., 2009; Becker et al., 2022). Their final integration usually depends on multicriteria decisional analysis methods that require expert judgment for weighing the final diagnostic outcomes (e.g., Semenzin et al., 2008). Providing a unique method that accommodates all new diagnostic tools available for different sources of chemical ecosystem impairment and freshwater ecosystems is beyond the scope of this paper, but it seems that the individual uncertainties of some of the new diagnostic tools and indicators available would need to be reevaluated in this context. Figure 4 addresses this by proposing a qualitative framework to assess such uncertainties. Additionally, integrating emerging tools like AOPs, mixture toxicity models, and machine learning algorithms could refine uncertainty quantification and improve data synthesis for environmental decision-making (Kienzler et al., 2022; Bell et al., 2023).

## Conclusions

Multiple diagnostic tools have been developed during the last decades to provide diverse lines of evidence for assessing ecological risks under field conditions. These tools are designed to address the intricate interactions and impacts of chemical stressors, offering a more nuanced perspective on ecosystem health. As highlighted in this paper, each method evaluates distinct aspects of ecosystems and can be seen as highly complementary, contributing to a comprehensive understanding of the (net) effects of chemical pollution on aquatic ecosystems. However, the limited number of studies that have attempted to compare the outcomes of these methods (including this one) reveal inconsistencies. These may arise from factors such as variations in the availability and quality of toxicity data used in toxic pressure assessments, unknown (or not assessed) biotic and abiotic stressors in the respective samples, disciplinary biases driven by differing study needs, or challenges in extrapolating findings across different levels of biological organization, among others. Understanding these limitations is critical for interpreting chemical risk assessment results and may facilitate the hierarchical and complementary application of these tools. The integration of these diagnostic tools with AOPs, effect models, and machine learning algorithms, that help reduce the uncertainties in extrapolations across ecosystem receptors, levels of biological organization and ecological scenarios is expected to enhance the reliability of ecological risk assessments in the future.

## Supplementary information

Supporting information

## Data Availability

The data analysed in this study is provided in the Supplementary Information file.
